# Association between stress-related disorders and the risk of dementia using the Korean National Sample Cohort: a matched cohort study

**DOI:** 10.1038/s41598-023-43884-3

**Published:** 2023-10-01

**Authors:** Hyunkyu Kim, Yu Shin Park, Seung Hoon Kim, Kyungduk Hurh, Jinhyun Kim, Eun-Cheol Park, Sung-In Jang

**Affiliations:** 1 Department of Preventive Medicine, Institute of Health Services Research, Yonsei University College of Medicine, 50 Yonsei-ro, Seodaemun-gu, Seoul, 03722 Republic of Korea; 2 Institute of Health Services Research, Yonsei University, Seoul, Republic of Korea; 3 Department of Psychiatry, Yonsei University College of Medicine, Seoul, Republic of Korea; 4 Department of Public Health, Graduate School, Yonsei University, Seoul, Republic of Korea; 5 Department of Preventive Medicine, Eulji University School of Medicine, Daejeon, Republic of Korea

**Keywords:** Post-traumatic stress disorder, Dementia

## Abstract

Post-traumatic stress disorder (PTSD) is associated with the development of dementia; however, the association of dementia risk with overall stress-related disorders is less known. This study investigated the association between stress-related disorders and the risk of dementia in a Korean nationwide sample cohort. The data analyzed in this study were acquired from the Korean National Health Insurance Service National Sample Cohort between 2002 and 2013. Using a 1:3 propensity score matching, 8906 patients with stress-related disorders and 26,718 control participants were included in the analysis. Patients with stress-related disorders had a higher risk of developing dementia after adjusting for covariates (hazard ratio [HR] = 1.15; 95% confidence interval [CI] 1.01–1.30) than control participants. Patients with PTSD showed the highest risk of increase (HR = 1.78) than those with other types of stress-related disorders. Patients with stress-related disorders showed the highest and significantly increased risk for Alzheimer’s dementia (HR = 1.22, 95% CI 1.04–1.56). These results indicated an association between a history of stress-related disorders and the risk of dementia in the South Korean population. Further research investigating the causal mechanisms is needed.

## Introduction

Stress has a diverse impact on individuals, affecting them both physically and mentally with either acute or chronic effects^1,2^. Although some individuals may overcome such stress effectively and quickly, in others, stress can have a prolonged and significant impact, leading to symptoms such as re-experiencing and hyperarousal. Ultimately, these individuals may be diagnosed with stress-related disorders^3^. Some studies suggest that exposure to stress can result in irreversible damage or changes in the brain, and even cause degenerative changes^4,5^. These degenerative changes in the brain could lead to the onset of dementia, and previous studies have demonstrated that post-traumatic stress disorder (PTSD), which is considered the most severe form of stress-related disorders, is associated with an increased risk of developing dementia later in life^6–8^. Patients with PTSD had twice the risk for developing dementia (HR = 2.31) compared with patients without PTSD among US veterans^7^. However, previous studies have primarily focused on the relationship between PTSD and dementia, thereby limiting the evidence regarding the association between dementia and other stress-related disorders that may be milder in form or shorter in duration than PTSD. If acute stress disorders or any milder forms like adjustment disorders are also associated with dementia, it would suggest that appropriate treatment is necessary even in such cases to avoid the potential link to future dementia risk. Furthermore, previous studies have generally focused on military veterans, who might substantially differ from the general population in terms of the types of stress they face^7,9,10^. There have also been only a few studies conducted on non-veteran populations^11,12^, so we believe there is a need to confirm whether such an association between stress-related disorders and dementia exists in a large sample of the general population. The lifetime prevalence of PTSD in Korea is 1.5%, and the annual incidence of acute stress disorder and PTSD had increased between 2011 and 2017.^13,14^ Furthermore because of rapid aging, the prevalence of dementia has quickly risen from 5.9% in 2015 to 7.3% in 2019.^15^ Therefore, we believe that research on the association between these conditions is necessary in the general population of Korea.

This study aimed to investigate the association between stress-related disorders and the risk of dementia in a representative sample of Koreans using nationwide claims cohort data. We further aimed to investigate whether the association differs among stress-related disorder types, including PTSD and adjustment disorder, and dementia types, such as Alzheimer’s disease and vascular dementia.

## Methods

### Study population and data

The data analyzed in this study were acquired from the Korean National Health Insurance Service National Sample Cohort (NHIS-NSC) between 2002 and 2013^16^. The Korean NHIS provides researchers with all data on claims collected under the NHIS for academic investigation and policymaking. The NHIS-NSC data include all medical claims from 1,025,340 individuals, accounting for 2% of the South Korean population by random sampling. Patients in the cohort were followed up unless they were excluded because of death or migration. The NHIS-NSC database includes information on socioeconomic status (SES) and clinically determined International Classification of Diseases, 10^th^ revision (ICD-10) codes.

Patients who sought treatment for stress-related disorders or dementia within the washout period of 1 year were excluded as this study aimed to investigate patients with newly developed stress-related disorders and dementia^16^. Moreover, patients diagnosed with dementia within 1-year of the stress-related disorders diagnosis were excluded from the study to exclude patients who potentially already had both diseases. A total of 1636 individuals were excluded for having been diagnosed with dementia prior to their stress-related disorder, developing dementia within 1 year following their stress-related disorder diagnosis, or receiving a diagnosis of stress-related disorder or dementia in 2002. Additionally, 12,759 individuals were excluded for being under the age of 40. After exclusion, 8906 patients were included in the stress-related disorder group.

Patients in the stress-related disorder group were 1:3 matched with those in the control group who had not been previously diagnosed with stress-related disorders or dementia via propensity score matching. The propensity score was derived using logistic regression to calculate the probability of dementia, with covariates of sex, age, insurance, and income status. After calculating the propensity score, one to three greedy matching on propensity scores was performed using the OneToManyMTCH macro in SAS^17^.

### Study variables and covariates

The patients were diagnosed with stress-related disorders based on the diagnostic ICD-10 code F43 at the first visit. Patients with ICD-10 codes for Alzheimer’s disease (F00, G30), vascular dementia (F01), and other types of dementia (F02, F03, G31.00, G31.82) were considered to have dementia. For each patient, we identified the date of diagnosis of stress-related disorders or the end of the study period (December 30, 2013) as the final follow-up date. We included various patient-related variables that may affect the occurrence of stress-related disorders or dementia, such as age, sex, social security status, residential region, disability, income status, baseline depressive disorder history, baseline anxiety disorder history, and the Charlson comorbidity index (CCI), which were included in the regression model as covariates^16,18^ CCI was used to identify the patients’ comorbidities, which were calculated by weighting and scoring for comorbid diseases. Patients were divided into three groups according to CCI (0–2, 3–4, and ≥ 5). Patients were divided into four groups based on age (10-year groups; 40–49, 50–59, 60–69, and ≥ 70 years) to identify the difference in dementia risk among the age groups. The region was divided into the following three categories according to population density: metropolitan, urban, and rural. Social security status was categorized by patients’ health insurance status based on the criteria of South Korea's National Health Insurance system. Patients who are self-employed or employed by a company are covered by National Health Insurance. Medical aid beneficiaries are individuals who have an income below the government-defined poverty threshold or disability, enabling them to receive free inpatient- and outpatient care from the government.

### Statistical analysis

We first examined the frequencies and percentages of each categorical variable for each patient at baseline and performed chi-square tests to examine the distribution of dementia diagnosis according to each variable^16^. Consequently, a Cox proportional hazards model was generated to examine the association between stress-related disorders and the risk of dementia. Covariates, including SES, were included in the analysis. Independent subgroup analyses were performed to investigate the combined effects of stress related disorder and other covariates on dementia. The results are presented as hazard ratios (HRs) and 95% confidence intervals (95% CIs) to compare the risk of dementia among the groups. All analyses were conducted using SAS software (version 9.4; SAS Institute, Cary, North Carolina, USA) and statistical significance was determined by a two-tailed test with a P-value of 0.05.

### Ethics approval and consent to participate

We assert that all procedures contributing to this work comply with the ethical standards of the relevant national and institutional committees on human experimentation and the Helsinki Declaration of 1975, as revised in 2008. Informed consent requirement was waived as the database we used in this study was based on routinely collected administrative and claims data. As the NHIS-NSC data did not contain any identifying information, the study was approved by the Institutional Review Board of Yonsei University’s Health System (4-2021-0208).

## Results

General characteristics of the study population and results of the univariate analyses are presented in Table 1. A total of 35,624 patients were included in the analysis, and 3.6% of them were diagnosed with dementia. A significant difference was observed in the prevalence of dementia between groups divided by the diagnosis of stress-related disorders. Other demographic variables were significantly associated with the occurrence of dementia during the study period (*P* < 0.0001).

**Table 1:** General characteristics of the study population and the results of the chi-square test with risk of dementia.

Variables	Total	Risk of dementia		*P* value
		Yes	No	
Total	35,624 (100.0)	1277 (3.6)	34,347 (96.4)	
Stress related disorders				< 0.0001
Yes	8906 (25.0)	411 (4.6)	8495 (95.4)	
No	26,718 (75.0)	866 (3.2)	25,852 (96.8)	
Gender				< 0.0001
Male	11,833 (33.2)	335 (2.8)	11,498 (97.2)	
Female	23,791 (66.8)	942 (4.0)	22,849 (96.0)	
Age				< 0.0001
40–49	17,823 (50.0)	82 (0.5)	17,741 (99.5)	
50–59	9672 (27.2)	216 (2.2)	9456 (97.8)	
60–69	6136 (17.2)	546 (8.9)	5590 (91.1)	
≥ 70	1993 (5.6)	433 (21.7)	1560 (78.3)	
Social security				< 0.0001
Health insurance	35,107 (98.5)	1,238 (3.5)	33,869 (96.5)	
Medical aid	517 (1.5)	39 (7.5)	478 (92.5)	
Region				< 0.0001
Metropolitan	14,767 (41.5)	438 (3.0)	14,329 (97.0)	
City	9277 (26.0)	328 (3.5)	8949 (96.5)	
Rural	11,580 (32.5)	511 (4.4)	11,069 (95.6)	
Disability				< 0.0001
Yes	1259 (3.5)	91 (7.2)	1168 (92.8)	
No	34,365 (96.5)	1186 (3.5)	33,179 (96.5)	
Income				< 0.0001
Low	14,767 (41.5)	438 (3.0)	14,329 (97.0)	
Middle	9277 (26.0)	328 (3.5)	8949 (96.5)	
High	11,580 (32.5)	511 (4.4)	11,069 (95.6)	
Depression History				< 0.0001
Yes	3915 (11.0)	208 (5.3)	3707 (94.7)	
No	31,709 (89.0)	1069 (3.4)	30,640 (96.6)	
Anxiety Disorder History				< 0.0001
Yes	6059 (17.0)	296 (4.9)	5763 (95.1)	
No	29,565 (83.0)	981 (3.3)	28,584 (96.7)	
Hypertension				< 0.0001
Yes	18,169 (51.0)	1008 (5.5)	17,161 (94.5)	
No	17,455 (49.0)	269 (1.5)	17,186 (98.5)	
Diabetes				< 0.0001
Yes	8966 (25.2)	532 (5.9)	8434 (94.1)	
No	26,658 (74.8)	745 (2.8)	25,913 (97.2)	
Charlson Comorbidity Index (CCI)				< 0.0001
0–2	13,863 (38.9)	149 (1.1)	13,714 (98.9)	
3–4	13,173 (37.0)	314 (2.4)	12,859 (97.6)	
≥ 5	8588 (24.1)	814 (9.5)	7774 (90.5)	

Table 2 shows the results of survival analysis using the Cox proportional hazards regression model to investigate the association between stress-related disorders and the risk of dementia after adjusting for the covariates included in Table 1. Individuals with stress-related disorder history showed 1.15-times higher risk of dementia risk after adjusting for covariates (HR = 1.15, 95% CI 1.01–1.30). The group with females showed a higher risk of dementia compared with that with males (HR = 1.21, 95% CI 1.07–1.38). The risk of dementia increased with increasing age (HRs of the 50 s, 60 s, and ≥ 70 s groups were 3.77, 12.98, and 31.55, respectively, compared with that of the 40 s group). The number of comorbidities was also significantly associated with the risk of dementia, with patients with a higher CCI showing a higher risk of dementia. (HR = 1.64 in CCI 3–4, HR = 4.10 in CCI ≥ 5).

**Table 2:** Results of the Cox proportional hazard regression analysis on the association between stress-related disorder and risk of dementia.

Variables	Risk of dementia	
	HR	95% CI
Stress related disorders		
Yes	1.15	(1.01–1.30)
No	1.00	
Gender		
Male	1.00	
Female	1.21	(1.07–1.38)
Age		
40–49	1.00	
50–59	3.77	(2.91–4.87)
60–69	12.98	(10.21–16.50)
≥ 70	31.55	(24.66–40.36)
Social security		
Health Insurance	1.00	
Medical aid	4.28	(3.02–6.08)
Region		
Metropolitan	1.00	
City	1.16	(1.01–1.34)
Rural	1.06	(0.93–1.20)
Disability		
Yes	1.55	(1.25–1.93)
No	1.00	
Income		
Low	1.00	
Middle	0.94	(0.80–1.12)
High	0.92	(0.78–1.08)
Depression history		
Yes	1.46	(1.24–1.71)
No	1.00	
Anxiety disorder history		
Yes	1.35	(1.17–1.55)
No	1.00	
Hypertension		
Yes	1.11	(0.96–1.28)
No	1.00	
Diabetes		
Yes	1.01	(0.90–1.13)
No	1.00	
Charlson Comorbidity Index (CCI)		
0–2	1.00	
3–4	1.64	(1.34–1.99)
≥ 5	4.10	(3.41–4.93)

Independent subgroup analyses were conducted to assess the combined effects of stress-related disorders and other sociodemographic variables on the risk of dementia (Table 3). The risk of dementia was higher in the younger age group than in the older age groups (HRs of the 40 s and ≥ 70 groups were 1.55 and 1.02, respectively). Patients with health insurance showed a significant risk of dementia when they had stress-related disorders (HR = 1.17, 95% CI = 1.03–1.33); however, patients with medical aid did not show a significant association between stress-related disorders history and dementia risk.

**Table 3:** Subgroup analysis of the association between the risk of dementia and covariates according to stress-related disorder history.

	No SRD		SRD
	Adjusted HR	Adjusted HR	95% CI
Gender			
Male	1.00	1.31	(1.03–1.66)
Female	1.00	1.09	(0.94–1.26)
Age			
40–49	1.00	1.55	(0.98–2.45)
50–59	1.00	1.16	(0.87–1.55)
60–69	1.00	1.19	(0.99–1.43)
≥ 70	1.00	1.02	(0.82–1.28)
Social security			
Health insurance	1.00	1.17	(1.03–1.33)
Medical Aid	1.00	0.47	(0.20–1.08)
Region			
Metropolitan	1.00	1.05	(0.85–1.30)
City	1.00	1.28	(1.01–1.61)
Rural	1.00	1.14	(0.93–1.39)
Income			
Low	1.00	1.04	(0.77–1.39)
Middle	1.00	1.17	(0.96–1.42)
High	1.00	1.18	(0.98–1.42)
Disability			
Yes	1.00	1.17	(1.03–1.33)
No	1.00	0.75	(0.45–1.24)
Depression history			
Yes	1.00	1.20	(0.90–1.58)
No	1.00	1.14	(0.99–1.30)
Anxiety disorder history			
Yes	1.00	1.17	(0.93–1.48)
No	1.00	1.13	(0.98–1.31)
Hypertension			
Yes	1.00	1.15	(1.00–1.31)
No	1.00	1.11	(0.83–1.47)
Diabetes			
Yes	1.00	1.05	(0.87–1.27)
No	1.00	1.22	(1.04–1.44)
Charlson Comorbidity Index (CCI)			
0–2	1.00	1.47	(0.97–2.20)
3–4	1.00	1.09	(0.84–1.42)
≥ 5	1.00	1.13	(0.97–1.31)

After subgrouping the stress-related disorders into four groups according to the ICD-10 code, patients with PTSD showed the highest risk of dementia (HR = 1.78, 95% CI 1.13–2.81) compared with those in other groups (Table 4). The subgroup of patients with adjustment disorder also showed a significantly elevated risk (HR = 1.32, 95% CI 1.05–1.65) for dementia. Among the dementia types, patients with stress-related disorders showed the highest and significantly increased risk for Alzheimer’s dementia (HR = 1.22, 95% CI 1.04–1.44, Table 5).

**Table 4:** Type of stress-related disorders and their association with dementia.

	Risk of dementia	
	HR	95% CI
No stress related disorder	1.00	
Acute stress reaction	1.20	(0.99–1.45)
Post-traumatic stress disorder	1.78	(1.13–2.81)
Adjustment disorders	1.32	(1.05–1.65)
Other reactions to severe stress		
Reaction to severe stress, unspecified	1.15	(0.97–1.35)

**Table 5:** The association between stress-related disorders and different types of dementia.

	Alzheimer’s dementia (n = 685)		Vascular demented (n = 167)		Other types of Dementia (n = 425)	
	HR	95% CI	HR	95% CI	HR	95% CI
No SRD	1.00		1.00		1.00	
SRD	1.22	(1.04–1.44)	1.11	(0.79–1.56)	1.05	(0.85–1.30)

## Discussion

This study aimed to investigate the association between stress-related disorders and the risk of dementia and whether the association differs among stress-related disorder and dementia types in a South Korean nationwide sample cohort over a 12-year follow-up period. We found that a history of stress-related disorders was associated with an increased risk of dementia. Furthermore, PTSD was the stress-related disorder type most significantly associated with a history of dementia, whereas and Alzheimer’s disease was the dementia type with the highest risk of increase in patients with stress-related disorders.

Our study results generally agreed with those of previous studies on the association between stress-related disorders and the risk of dementia. Previous studies have mainly focused on the development of dementia in patients with PTSD and showed that veterans with PTSD have approximately a two-fold higher risk of development of dementia after the follow-up period, which was similar to our result with an HR of 1.95^7,10,19–23^. Our findings are similar to those of previous studies, in which participants from the general population showed an association between PTSD and dementia risk^11,12,24^. One meta-analysis suggested that the risk of dementia in the general population was higher than that in veterans^6^. Only few studies have focused on a full range of stress-related disorders, the results of which suggested the association of stress-related disorders with dementia, which is similar to the results of our study^11,25^.

Our study indicated that not only PTSD but also other types of stress-related disorders were associated with the development of dementia. Among the types of stress-related disorders, PTSD is associated with the highest risk of dementia. This result showed that the most severe and long-lasting type of stress-related disorder has the strongest association with the risk of dementia, suggesting that stronger stresses may increase the risk of dementia. Adjustment disorder also showed a significantly elevated risk for dementia, indicating that PTSD is not the sole contributor to increased dementia risk observed in stress-related disorders. The primary distinction between PTSD and adjustment disorder is the stress or trauma intensity and consequent reaction to it^26^. Although the stressors leading to PTSD are often sudden or life-threatening, those causing adjustment disorder are typically less severe and not life-threatening. As a result, the symptoms manifest differently from those seen in PTSD^27^. Therefore, if stressors capable of causing adjustment disorder are associated with the onset of dementia, caution is warranted even for patients with such disorders. However, the dementia risk from PTSD is higher than that from adjustment disorder, which is presumed to result from a more intense stressor^28^. Previous studies have shown that the risk of dementia increases with higher perceived stress, which likely supports this hypothesis^29^. However, in our study, we used diagnostic codes data instead of actually assessing the stressors. Thus, pinpointing the exact reason remains challenging and further research is needed.

In our subgroup analysis, HR decreased inversely with age, and notably, almost no increase in risk was observed for individuals aged 70 years and above. This could be because increased age is a significant risk factor for dementia, thereby relatively diminishing the risk elevation attributable to stress-related disorders. In Table 2, the risk for dementia in participants aged 70 years and above was 31.55 times higher compared with those in their 40 s; previous studies have also considered higher age as a significant risk factor for dementia^30–32^. This suggests that young people who experience stress-related disorders could be at a higher risk of developing dementia later in life, highlighting the need for long-term follow-up and management. When conducting a subgroup analysis based on social security, income, and disability, the results showed a tendency for the HR to decrease in groups with lower SES. A possible reason for this could be that, similar to age, a lower SES may be a significant risk factor for dementia. In Table 2 of our study, a lower SES was associated with a higher risk of dementia, which supports a similar previous finding^33^. Furthermore, patients with lower SES may be less likely to seek medical care for stress-related disorders. Because our analysis was based on healthcare claims data for individuals who visited psychiatric clinics rather than direct assessments, there is a potential for underestimation owing to people who did not visit a hospital. Previous research analyzing hospital visits for PTSD based on national income levels found that people in high-income countries are twice as likely to seek treatment compared with those in low-income countries^34^, suggesting that low SES might be associated with the low visiting rate of clinic due to the PTSD.

The mechanisms underlying the association between stress-related disorders and the development of dementia remain unclear. One hypothesis is based on previous evidence of the association between stress-related disorders and cardiovascular disease, suggesting that dementia may develop via vascular impairment^35–37^. In our study, the risk of vascular dementia among patients with stress-related disorders was increased but not significantly, which may be because of the small sample size and short follow-up period. Another hypothesis states that certain neurobiological pathways induced by stress, which alter the hypothalamic–pituitary–adrenal axis, may affect the hippocampus. This impact may reduce hippocampal volume and induce oxidative damage in the hippocampus, which in turn accelerates the process of hippocampus degeneration^38–40^. Hippocampal atrophy is a characteristic of Alzheimer’s disease, for which the risk of development was the highest in patients with stress-related disorders. Another hypothesis is that low resilience may act as a common underlying factor linking stress-related disorders and dementia. Resilience is the ability to adapt successfully when faced with significant adversity, and individuals with low resilience are at a higher risk of developing stress-related disorders, including PTSD^41,42^. Individuals with high resilience have a lower risk of clinical dementia or experience its onset at a later stage^43^. Thus, low resilience to stress might be the common risk factor for stress-related disorder and dementia. Further investigation on the mechanism should be conducted to prevent dementia development among patients with stress-related disorders.

Our study had several limitations. First, this was a retrospective cohort study and could not include all variables that might affect the development of dementia. Family history, health behaviors including smoking and drinking, and laboratory data were not included in the analysis. Second, the number of patients diagnosed with stress-related disorders may have been underestimated in the analysis. The number of actual stress-related disorder patients might be higher than the number we included in our analysis because people hesitate to visit psychiatric clinics in South Korea. Third, the claims data provided only the diagnostic codes for claim; thus, the accuracy might be limited, as suggested in a previous study^44^. To enhance the accuracy of diagnosis, we included the primary and secondary diagnostic codes of each visit in the analysis. Fourth, we could not evaluate the severity of stress-related disorders and dementia symptoms. As we only defined the patients by the claimed ICD-10 code and could not use the scale for each disorder, we could not assume the severity of symptoms. Fifth, our study did not reflect for patients with mixed diagnoses or those whose diagnoses changed over time. Further, because we were unable to directly assess the symptoms of patients and relied on health insurance claims data, there is the possibility that not all changes were recorded. To minimize the instability of the diagnoses, we opted to use the diagnoses given at the patients' first visit, which are likely to be relatively stable and reflect the initial symptoms and severity of the patients. Finally, owing to the retrospective nature of the study, causal relationships could not be established. The increased risk of dementia may be the result of a temporal relationship rather than a causal relationship.

Despite these limitations, this study had several strengths. Although the data we used has several limitations due to the characteristics of claims data, the data represents the general population of South Korea because these are sample cohort from the entire population. Therefore, the results of our study can be generalized to the entire Korean population or other countries with similar population structure and may provide a background for the management of dementia occurrence among patients with stress-related disorders. We also applied propensity score matching for exposure variables; thus, confounding factors between patients with and without stress-related disorders were reduced, and the comparability was enhanced.

## Conclusions

This study identified an association between the history of stress-related disorders and risk of dementia in a South Korean national representative cohort. Individuals diagnosed with stress-related disorders are at 1.15-times higher risk of dementia. Furthermore, among the types of stress-related disorders, PTSD was associated with the highest risk of dementia development, and adjustment disorder also increased the risk of dementia. Alzheimer’s disease was the dementia type with the highest risk of increase among individuals with stress-related disorders. Further research using a prospective design to clarify the causality or mechanisms of the relationship between stress-related disorders and dementia in a controlled environment should be conducted to validate these findings.

## Data Availability

The Korean National Health Insurance Service–National Sample Cohort is a public, open-access database. It is based on the health insurance claim data of all Koreans, and the sample cohort is available for public purposes and scientific research. The sample cohort data are available after acceptance of approval for use by the national health insurance service (https://nhiss.nhis.or.kr/bd/ab/bdaba000eng.do).
